# Fermentation-driven microbial and metabolic shifts in filler tobacco leaves of different grades

**DOI:** 10.3389/fmicb.2025.1651289

**Published:** 2025-09-24

**Authors:** Chen He, Shaoxin Yang, Shengnan Dong, Shengxiao Wang, Pengfei Zhang, Yang Yang, Delong Xu, Rongchao Yang, Bo Zeng, Yanqi Hu, Qing Zhang

**Affiliations:** ^1^Zhengzhou Tobacco Research Institute of China National Tobacco Corporation, Zhengzhou, China; ^2^China Tobacco Shandong Industrial Co Ltd., Jinan, China

**Keywords:** filler tobacco leaves, fermentation, metagenomics, untargeted metabolomics, quality grading

## Abstract

**Introduction:**

Filler tobacco leaves (FTLs) serve as the primary raw material for cigar production, and notable differences in physicochemical properties and fermentation responsiveness exist across different grades. However, the underlying mechanisms governing microbial and metabolic evolution during FTL fermentation remain poorly understood. This study systematically investigated the microbial community structures and metabolomic profiles of FTLs of varying grades before and after fermentation using metagenomic sequencing and untargeted metabolomics.

**Results:**

Metagenomic analysis revealed marked differences in microbial composition among FTL grades at the onset of fermentation. The fermentation process further facilitated the enrichment of functional genera such as *Bacillus*, *Escherichia*, and *Alternaria*, while low-grade FTLs exhibited excessive accumulation of *Corynebacterium*, potentially contributing to off-flavors and undesirable odors. Untargeted metabolomics identified numerous significantly differential metabolites after fermentation, primarily enriched in pathways related to amino acid biosynthesis, sugar metabolism, and carotenoid biosynthesis. Principal component analysis and hierarchical clustering indicated partial continuity in metabolomic profiles within the same grade before and after fermentation. Correlation analysis further revealed strong positive associations between several dominant genera and flavor-related metabolites.

**Conclusion:**

This study demonstrates that FTLs of different grades exhibit distinct patterns of microbial succession and metabolic remodeling during fermentation. The initial leaf grade plays a pivotal role in shaping microbial communities and metabolite accumulation. These findings offer mechanistic insights into the fermentation process of FTLs and provide theoretical and practical guidance for optimizing raw material grading and fermentation management in the cigar industry.

## 1 Introduction

Cigar tobacco is a distinctive and economically important type of tobacco that differs significantly from cigarette tobacco in terms of processing methods and product attributes. Its specialized craftsmanship and unique flavor profile are highly appreciated by a niche group of consumers. As the primary raw material for cigar production, the quality of cigar tobacco leaves directly influences the smoking experience, combustibility, and storage stability of the final product (Zhang et al., 2025a). Based on their position on the plant and functional roles, cigar tobacco leaves are categorized into wrapper, binder, and filler tobacco leaves (FTLs). Among these, FTLs account for approximately 75% of a cigar’s total weight and serve as the structural core and main carrier of flavor, playing a crucial role in shaping the overall aroma profile (Zhang et al., 2024a).

Fermentation is a particularly critical stage in the traditional processing of cigar tobacco. Unlike the high-temperature flavoring treatments used for cigarette tobacco, cigar tobacco fermentation relies on gentle biochemical reactions mediated by naturally occurring microbial activity (Ren et al., 2024). Typically, two fermentation stages are involved: agricultural fermentation, which primarily aims to eliminate green odors and bitterness and to balance the chemical composition of raw leaves (Zhang et al., 2025b); and industrial fermentation, which further refines the product through targeted strategies to achieve desired sensory characteristics (Hu et al., 2023, 2025). During this process, intrinsic compounds such as sugars, polyphenols, proteins, and alkaloids undergo complex degradation, transformation, and recombination under the synergistic action of microorganisms and enzymes, ultimately determining the physicochemical characteristics and sensory quality of cigar tobacco leaves (Chen et al., 2025).

In recent years, advances in high-throughput sequencing and metabolomics technologies have enabled detailed investigations into the dynamic changes of microbial communities during fermentation and their roles in shaping tobacco chemistry (Xing et al., 2023; Song et al., 2024; Wang et al., 2024). Fermentation of FTLs is characterized by temporal succession of bacterial and fungal communities, accompanied by multilayered metabolic regulation such as the biosynthesis of aroma precursors, redox reactions, and the neutralization of alkaline substances (Tao et al., 2022; Si et al., 2023). Dominant genera including *Pseudomonas*, *Bacillus*, *Enterobacter*, and *Sphingomonas* initiate carbohydrate and protein degradation and contribute to the production of alcohols, esters, ketones, and other aroma-active compounds (Liu et al., 2021; Ren et al., 2024). Some studies have further emphasized that fermentation time strongly drives microbial succession (Tao et al., 2024), functional shifts in microbial taxa are associated with improved cigar-leaf quality (Liu et al., 2021), and key metabolic pathways are significantly remodeled during fermentation (Li et al., 2020). Collectively, these findings demonstrate the close linkage between microbial activity and metabolic remodeling in tobacco leaves. Nevertheless, the structural features, successional patterns, and functional roles of microbial communities in FTLs—particularly the differences among FTLs of varying grades—remain insufficiently understood.

In industrial practice, FTLs of different grades often display pronounced differences in their initial physicochemical properties and fermentation responses, influenced by factors such as leaf position, physiological maturity, cultivation practices, and pre-processing methods (Tao et al., 2024). Higher-grade FTLs generally exhibit greater fermentation adaptability and aromatic potential, whereas lower-grade FTLs are more susceptible to issues such as incomplete fermentation and the accumulation of off-flavor compounds (Zhang et al., 2025a). These disparities may arise not only from differences in chemical composition but also from variations in the initial structure and functional potential of microbial communities (Zhang et al., 2024a). Moreover, the grade-dependent shifts in metabolite profiles and the enrichment of specific metabolic pathways before and after fermentation merit in-depth investigation to elucidate the key mechanisms underlying fermentation quality differences.

Building on this foundation, the present study focused on “Yunxue No. 1,” a high-quality cigar tobacco variety independently developed in China. FTLs of high, medium, and low grades were selected following standardized cultivation, air-curing, and agricultural fermentation, and the industrial fermentation process was simulated under controlled temperature and humidity conditions. Metagenomic sequencing was conducted to characterize the dynamics of microbial communities before and after fermentation, while untargeted metabolomics was applied to analyze metabolite profiles, enriched pathways, and potential microbe–metabolite interactions. Unlike previous studies that primarily examined general fermentation processes or single leaf grades, this work systematically compares three distinct FTL grades, integrating shotgun metagenomics with untargeted metabolomics to reveal grade-dependent microbe–metabolite co-evolution. By elucidating how microbial communities and metabolite profiles evolve across different grades, this study identifies key biological factors influencing fermentation quality and advances the theoretical understanding of microbial ecology and metabolic regulation in cigar tobacco. The findings provide novel scientific evidence and practical guidance for raw material grading, fermentation process optimization, and flavor quality control in the cigar industry.

## 2 Materials and methods

### 2.1 Sample collection and preparation

The FTLs used in this study were obtained from “Yunxue No. 1,” a high-quality cigar tobacco variety independently developed in China. All samples were collected from the same production region and underwent standardized air-curing and agricultural fermentation. Leaf grading was performed by the collaborative partner, China Tobacco Shandong Industrial Co Ltd., in accordance with the “YC/T 588—2021 Industrial and commercial grade standards for cigar leaves.” Following a fermentation protocol similar to that described by Chen et al. (2025), technical guidance for industrial fermentation was provided by staff from China Tobacco Shandong Industrial Co Ltd. Prior to industrial fermentation, the raw FTLs were reconditioned to a uniform moisture content of approximately 35%. Subsequently, three representative grades—high (Fi-X-1-Bt-M), medium (Fi-X-2-Bt-M), and low (Fi-X-3-Bt-M)—were selected and designated as X1, X2, and X3, respectively. These samples were subjected to fermentation for 7 days under controlled conditions (40 ± 1 °C and 80 ± 2% relative humidity) in a temperature- and humidity-controlled incubator. The corresponding post-fermentation samples were designated as FX1, FX2, and FX3. All samples were flash-frozen in liquid nitrogen, ground into a fine powder, and stored at −80 °C for subsequent metagenomic and untargeted metabolomic analyses.

### 2.2 Metagenomic sequencing analysis

Metagenomic sequencing was conducted with reference to the method described by Tao et al. (2024), with appropriate modifications. Briefly, 10 g of frozen, finely ground FTL powder was mixed with 100 mL of 0.1 mol/L phosphate-buffered saline (PBS, pH 7.4; Macklin, China) and shaken at 180 rpm for 2 h to release surface-associated microorganisms. The resulting suspension was filtered through a 0.22 μm pore-size membrane, and microbial cells retained on the membrane were eluted with 40 mL of PBS. Plant debris in the eluate was removed using sterile gauze, and the filtered liquid was centrifuged at 12,000 × *g* for 15 min at 4 °C to collect microbial pellets. DNA extraction, quality assessment, library construction, and subsequent bioinformatics analyses were performed by Beijing Novogene Co., Ltd. Each sample group included three biological replicates.

### 2.3 Untargeted metabolomics analysis

A total of 100 mg of tissue sample, previously ground under liquid nitrogen, was placed into a 2 mL centrifuge tube and mixed with 500 μL of pre-chilled 80% (v/v) methanol (HPLC grade, Thermo Fisher Scientific, USA). The mixture was vortexed for 30 s and then incubated in an ice-water bath for 5 min. It was subsequently centrifuged at 15,000 × *g* for 20 min at 4 °C using a D3024R high-speed refrigerated centrifuge (Scilogex, USA). Then, 200 μL of the supernatant was transferred to a new tube and diluted with ultrapure water (HPLC grade, Merck, Germany) to adjust the final methanol concentration to 53% (v/v). After another 30 s of vortexing and a second centrifugation under the same conditions, the supernatant was filtered through a 0.22 μm organic-phase microporous membrane and subjected to ultra-high-performance liquid chromatography–tandem mass spectrometry (UHPLC–MS/MS) analysis (Want et al., 2013).

UHPLC–MS/MS analyses were performed on a Vanquish UHPLC system (Thermo Fisher Scientific, Germany) coupled to an Orbitrap Q Exactive™ HF or Q Exactive™ HF-X mass spectrometer (Thermo Fisher Scientific, Germany) at Beijing Novogene Co., Ltd. Each group contained six biological replicates. Chromatographic separation was achieved on a Hypersil GOLD C18 column (100 × 2.1 mm, 1.9 μm; Thermo Fisher Scientific, USA) using a 12-min linear gradient at a flow rate of 0.2 mL/min. The mobile phases consisted of solvent A (LC–MS grade water containing 0.1% formic acid; Thermo Fisher Scientific, USA) and solvent B (HPLC-grade methanol; Thermo Fisher Scientific, USA). The gradient program was set as follows: 0–1.5 min, 2% B; 1.5–3.0 min, linear increase to 85% B; 3.0–10.0 min, further increase to 100% B; 10.0–10.1 min, rapidly returned to 2% B; and 10.1–12.0 min, re-equilibration at 2% B.

The Orbitrap mass spectrometer was operated in both positive and negative electrospray ionization (ESI) modes with the following parameters: spray voltage, 3.5 kV; capillary temperature, 320 °C; sheath gas flow, 35 psi; auxiliary gas flow, 10 L/min; S-lens RF level, 60; and auxiliary gas heater temperature, 350 °C. To maximize metabolite coverage, raw data were collected under both ESI + and ESI– polarities and subsequently merged during data processing to generate a combined dataset for statistical analyses. MS/MS acquisition was performed in data-dependent acquisition (DDA) mode, which was selected to obtain high-quality and clean fragmentation spectra for reliable metabolite identification.

### 2.4 Data analysis

Metagenomics. For metagenomic sequencing data, raw reads generated on the Illumina NovaSeq platform were first quality-filtered using fastp to remove low-quality sequences and generate high-quality clean reads for downstream analysis. The clean data were then assembled using MEGAHIT, and the resulting scaffolds were split at ambiguous bases (N) to obtain scaffold contigs (scaftigs). Scaftigs longer than 500 base pairs (bp) were selected for open reading frame (ORF) prediction using MetaGeneMark, and predicted ORFs shorter than 100 nucleotides (nt) were discarded. To avoid interference from host sequences, clean reads were aligned against the tobacco reference genome, and all host-derived sequences were removed prior to downstream analysis. Non-redundant unigenes were annotated for taxonomic and functional information by aligning them against the MicroNR and the Kyoto Encyclopedia of Genes and Genomes (KEGG) databases using DIAMOND.

Untargeted metabolomics. For untargeted metabolomics data, raw mass-spectrometry files were processed using Compound Discoverer 3.3 (Thermo Fisher Scientific) to extract features such as retention time, mass-to-charge ratio (m/z), and peak intensity. Peak integration was based on the area under the curve, and molecular formulas were predicted by evaluating precursor and fragment ions. Metabolites were identified and relatively quantified by comparison with the mzCloud, mzVault, and MassList databases. Further metabolite annotation was conducted using KEGG, the Human Metabolome Database (HMDB), and LIPID MAPS.

Statistics and visualization. Multivariate statistical analyses were conducted to evaluate differences among sample groups. Principal coordinates analysis (PCoA) and non-metric multidimensional scaling (NMDS) based on Bray–Curtis dissimilarity were used to assess clustering patterns in terms of taxonomic and functional composition. Significant microbial taxa and functional pathways were identified using the Kruskal–Wallis and Wilcoxon rank-sum tests, followed by linear discriminant analysis (LDA) to evaluate effect size and perform dimensionality reduction. For metabolomics data, differences in metabolite abundance between groups were assessed using Student’s t-test (*p*-values), and fold change (FC) values were calculated. Principal component analysis (PCA) was performed based on correlation matrices, and hierarchical clustering was conducted using Pearson correlation coefficients and Euclidean distances. All figures and visualizations were generated using OriginPro 2024 and the Chiplot online platform.^1^

## 3 Results

### 3.1 Changes in microbial diversity and community composition before and after fermentation

To compare microbial community differences among FTLs of different grades, we first assessed alpha diversity using the ACE, Chao1, Shannon, and Simpson indices (Table 1). Prior to fermentation, the medium-grade group (X2) exhibited significantly lower values across all indices than the high- (X1) and low-grade (X3) groups (*p* < 0.05), indicating reduced microbial richness and a simpler baseline community structure in X2. By contrast, X1 and X3 displayed higher diversity, suggesting pronounced grade-dependent differences in initial community architecture.

**Table 1:** Alpha diversity indices of microbial communities in FTLs of different grades before and after fermentation.

Group	ACE	Chao1	Shannon	Simpson
X1	4890.78 ± 727.36^a^	4873.12 ± 728.31^a^	3.31 ± 0.31^ab^	0.81 ± 0.04^bc^
X2	2871.25 ± 196.64^c^	2868.26 ± 190.84^c^	2.96 ± 0.27^b^	0.77 ± 0.03^b^
X3	4815.27 ± 785.27^a^	4832.02 ± 830.76^a^	3.85 ± 0.45^a^	0.90 ± 0.04^ab^
FX1	5517.66 ± 163.08^a^	5538.40 ± 200.48^a^	3.87 ± 0.17^a^	0.94 ± 0.00^a^
FX2	2918.04 ± 496.59^c^	2909.98 ± 489.14^c^	3.13 ± 0.15^b^	0.79 ± 0.02^b^
FX3	3839.39 ± 273.10^b^	3887.51 ± 291.52^b^	1.82 ± 0.38^c^	0.50 ± 0.10^d^

Significant differences are denoted by different letters (one-way analysis of variance; *p* < 0.05).

Following fermentation, diversity changed in a grade-specific manner. In the high-grade group (FX1), alpha diversity indices increased, reflecting a richer and more even community. In the low-grade group (FX3), diversity declined sharply, with Shannon and Simpson decreasing to 1.82 and 0.50, respectively, consistent with pronounced community simplification.

**Figure 1:**
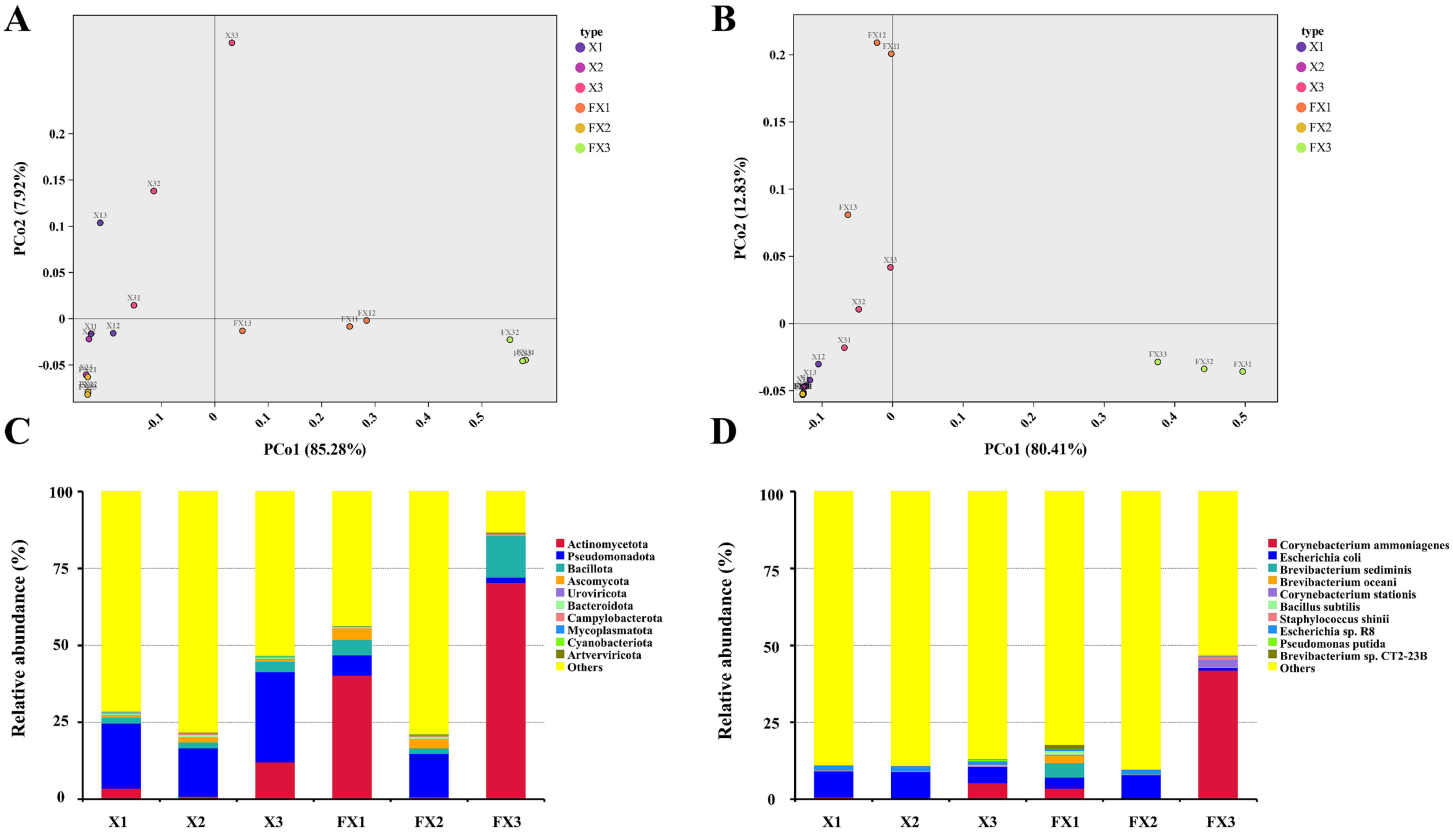
Beta diversity and community composition of microbial communities in FTLs of different grades before and after fermentation. Based on annotations from the MicroNR database, PCoA and relative abundance analyses were conducted at the phylum level **(A,C)** and species level **(B,D)** to illustrate the shifts in microbial community structure induced by fermentation. Note: “Others” indicates unclassified taxa after removal of tobacco host sequences.

Beta diversity and microbial composition patterns were further examined using PCoA and taxonomic profiling (Figure 1). At both the phylum and species levels, pre- and post-fermentation samples separated clearly, indicating a systemic restructuring of the microbiota. At the phylum level (Figure 1A), the PCo1 explained 85.28% of the variance, and FX3 showed the largest displacement along PCo1, suggesting the greatest compositional change. At the species level (Figure 1B), PCo1 and PCo2 together accounted for 92.74% of the variance; increased intergroup distances after fermentation indicate that the three grades followed distinct successional trajectories under identical processing conditions.

Taxonomic analyses highlighted differences in dominant phyla and species (Figures 1C,D). Before fermentation, Pseudomonadota and Actinomycetota predominated across all samples. After fermentation, Actinomycetota increased markedly in every group—exceeding 70% in FX3—while Pseudomonadota decreased, consistent with a trend toward compositional simplification. At the species level (Figure 1D), FX3 became dominated by *Corynebacterium ammoniagenes* (≈50% relative abundance), which was already detectable in X3 and proliferated rapidly during fermentation. In contrast, FX1 retained higher compositional complexity, including *Brevibacterium sediminis*, *Bacillus subtilis*, *Pseudomonas putida*, and *Escherichia coli*. Collectively, these data indicate that fermentation reshaped beta diversity and the dominance structure in a grade-dependent fashion: FX3 tended toward simplification, whereas FX1 maintained a comparatively complex community.

### 3.2 Functional prediction of microbial communities reveals differences in fermentation potential

**Figure 2:**
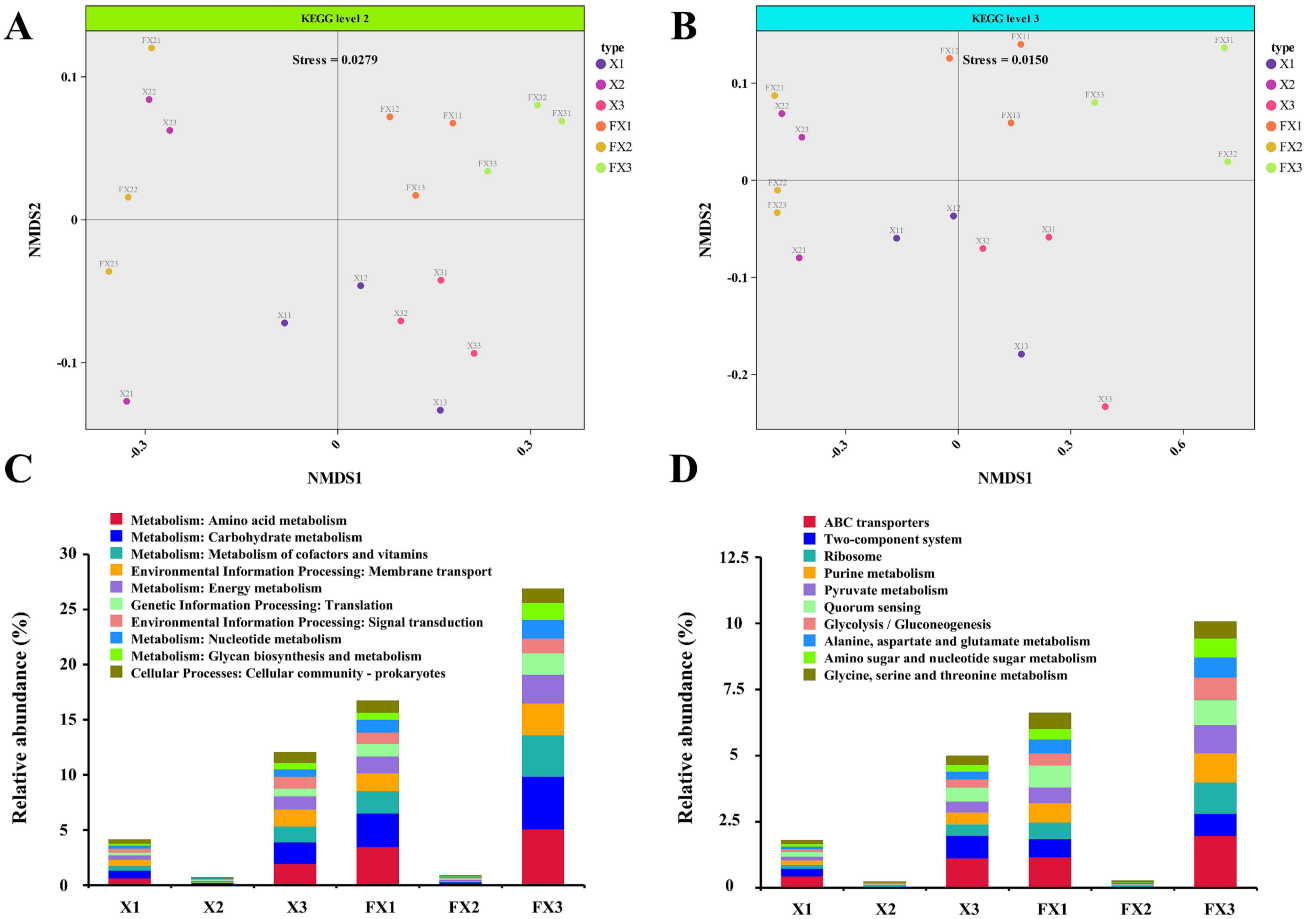
Functional profiling of microbial communities in FTLs of different grades before and after fermentation. **(A,B)** NMDS based on KEGG annotations showing differences in microbial functional composition at KEGG level 2 **(A)** and level 3 **(B)**; **(C,D)** Relative abundances of the top 10 functional pathways at KEGG level 2 **(C)** and level 3 **(D)** across samples before and after fermentation. Note: “Others” includes unannotated or unclassified functions after filtering host genome data.

As shown in Figure 2, the predicted functional profiles of the microbial communities differed markedly across FTL grades before and after fermentation. NMDS analysis (Figures 2A,B) revealed clear separation among samples in functional composition, with FX3 occupying the most distant position along both axes relative to the other groups, indicative of a more distinctive metabolic potential. The paired pre−/post-fermentation trajectories for each grade further illustrated fermentation-driven remodeling of microbial functions.

Functional abundance profiles derived from KEGG annotations resolved pathway-level differences among groups (Figures 2C,D). At KEGG level 2, the dominant categories included “amino acid metabolism,” “carbohydrate metabolism,” and “cofactor and vitamin metabolism” (Figure 2C). At level 3, enriched functions encompassed “ABC transporters,” “two-component systems,” “ribosome biosynthesis,” “purine metabolism,” and “pyruvate metabolism” (Figure 2D).

### 3.3 Differential biomarkers in microbial community and functional features

**Figure 3:**
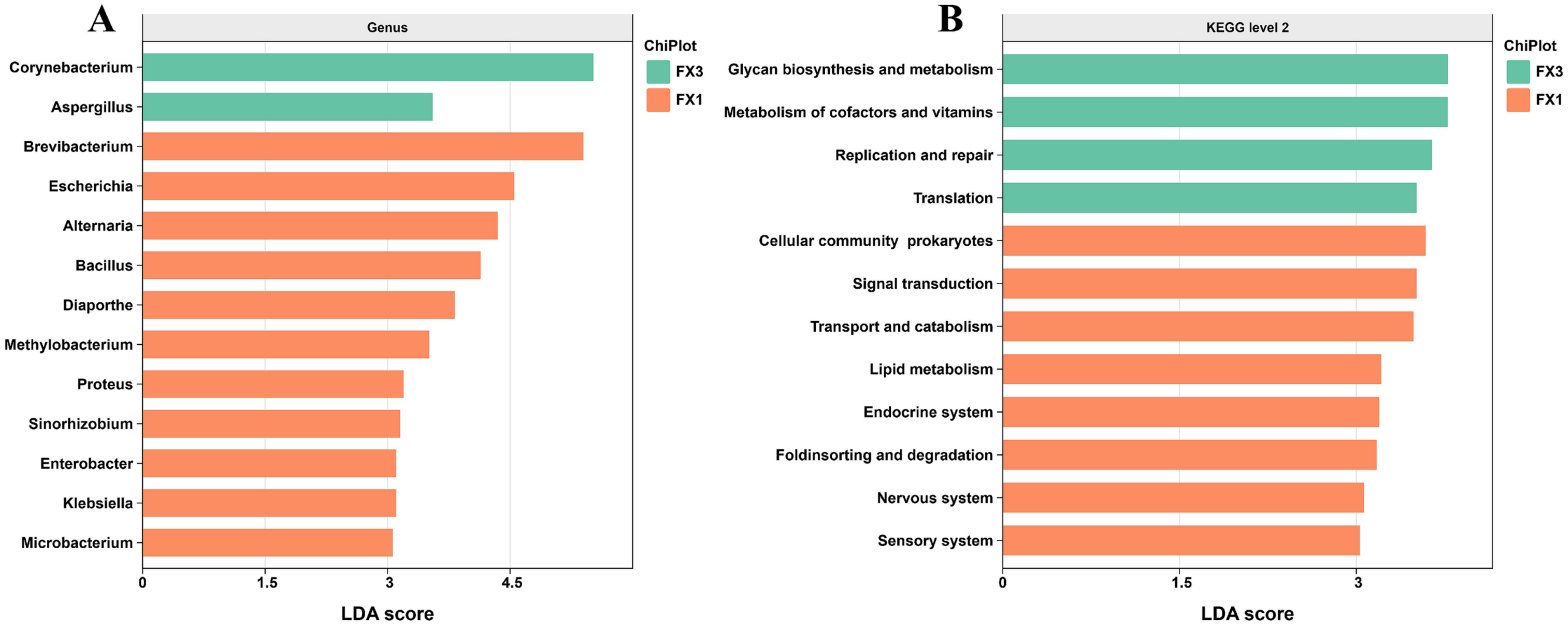
LEfSe analysis of differential microbial genera and functional pathways in high- and low-grade FTLs after fermentation. **(A)** Differential microbial genera identified at the genus level; **(B)** Differential functional pathways annotated at KEGG Level 2.

To further resolve the key post-fermentation differences in microbial structure and function between high- and low-grade FTLs, we performed LEfSe (linear discriminant analysis effect size) on the two groups with the most pronounced diversity contrast, FX1 and FX3. As shown in Figure 3A, at an LDA score threshold of ≥ 3, a total of 13 differential genera were identified. Among them, *Corynebacterium* and *Aspergillus* exhibited the highest LDA scores and were enriched in FX3, indicating greater representation of these taxa in the low-grade FTLs. By contrast, the remaining 11 genera—including *Brevibacterium*, *Escherichia*, *Alternaria*, *Bacillus*, and *Diaporthe*—were significantly enriched in FX1, consistent with a more diverse and feature-rich community in the high-grade FTLs.

Pathway-level contrasts (Figure 3B) provided the functional context for these structural differences. Microorganisms in FX3 were significantly enriched in “glycan biosynthesis and metabolism,” “metabolism of cofactors and vitamins,” “replication and repair,” and “translation.” In comparison, FX1 showed enrichment in “signal transduction,” “ABC transporters,” “transport and catabolism,” and “lipid metabolism.”

### 3.4 Metabolite classification characteristics

**Figure 4:**
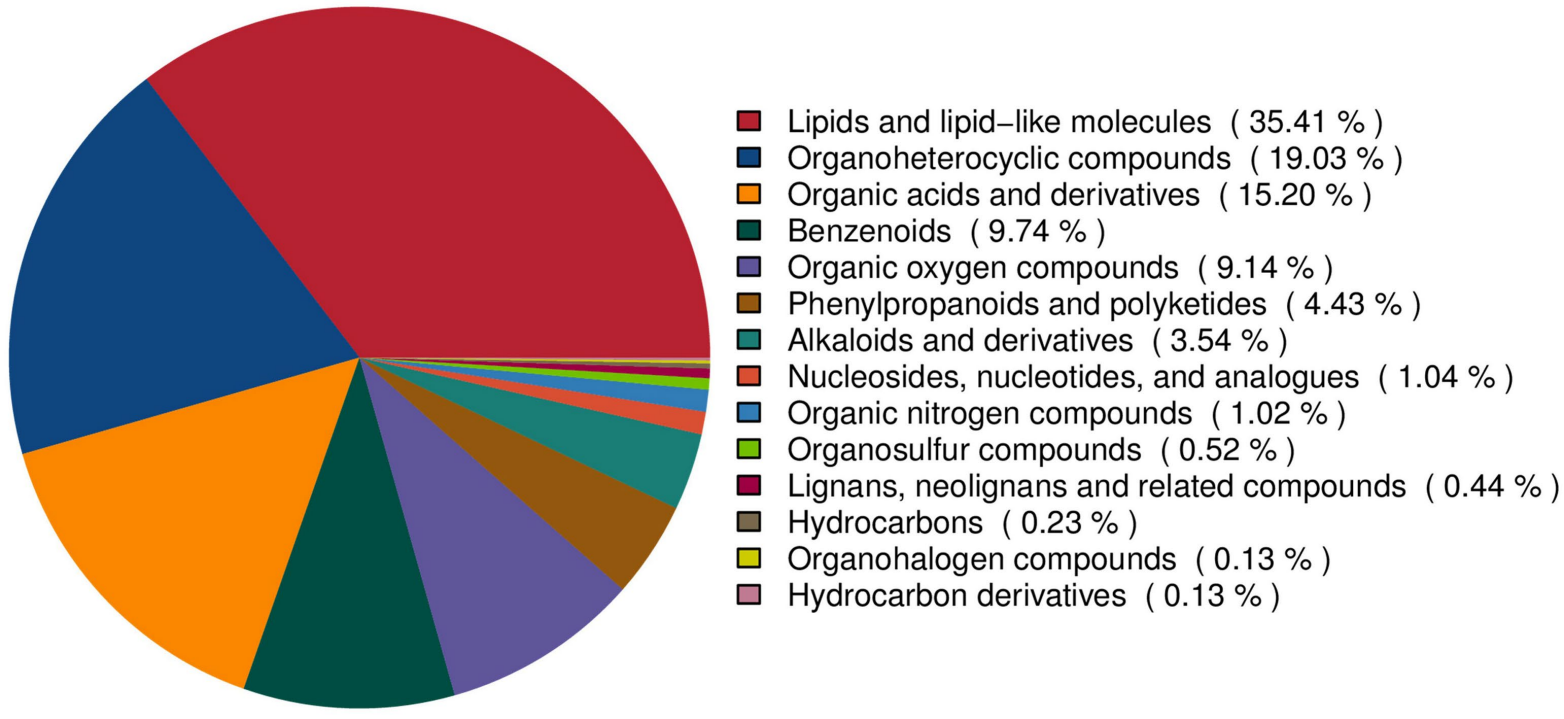
Chemical classification of metabolites detected in the metabolomic profiling of FTLs.

To comprehensively characterize the metabolite landscape during FTL fermentation, all detected features were classified by chemical ontology (Figure 4). The resulting profile encompassed multiple major classes, yielding a complex and chemically diverse metabolome. Lipids and lipid-like molecules constituted the largest proportion at 35.41%, representing the most abundant class in fermented FTLs. This was followed by organoheterocyclic compounds (19.03%) and organic acids and derivatives (15.20%). Benzenoids (9.74%) and organic oxygen compounds (9.14%) were also relatively abundant. In addition, smaller fractions of alkaloids and derivatives (3.54%), phenylpropanoids and polyketides (4.43%), nucleosides (1.04%), and other nitrogen-containing compounds (1.02%) were detected. Collectively, these data indicate that fermentation gives rise to a metabolite network predominantly composed of lipids, organic acids, aromatic compounds, and nitrogen-containing species. Compound-level qualitative identifiers (retention time, precursor m/z, major MS/MS fragments, adduct form, and acquisition polarity) and database annotations for all reported metabolites are provided in Supplementary Table S1.

### 3.5 Overall shifts in metabolic profiles of FTLs before and after fermentation

**Figure 5:**
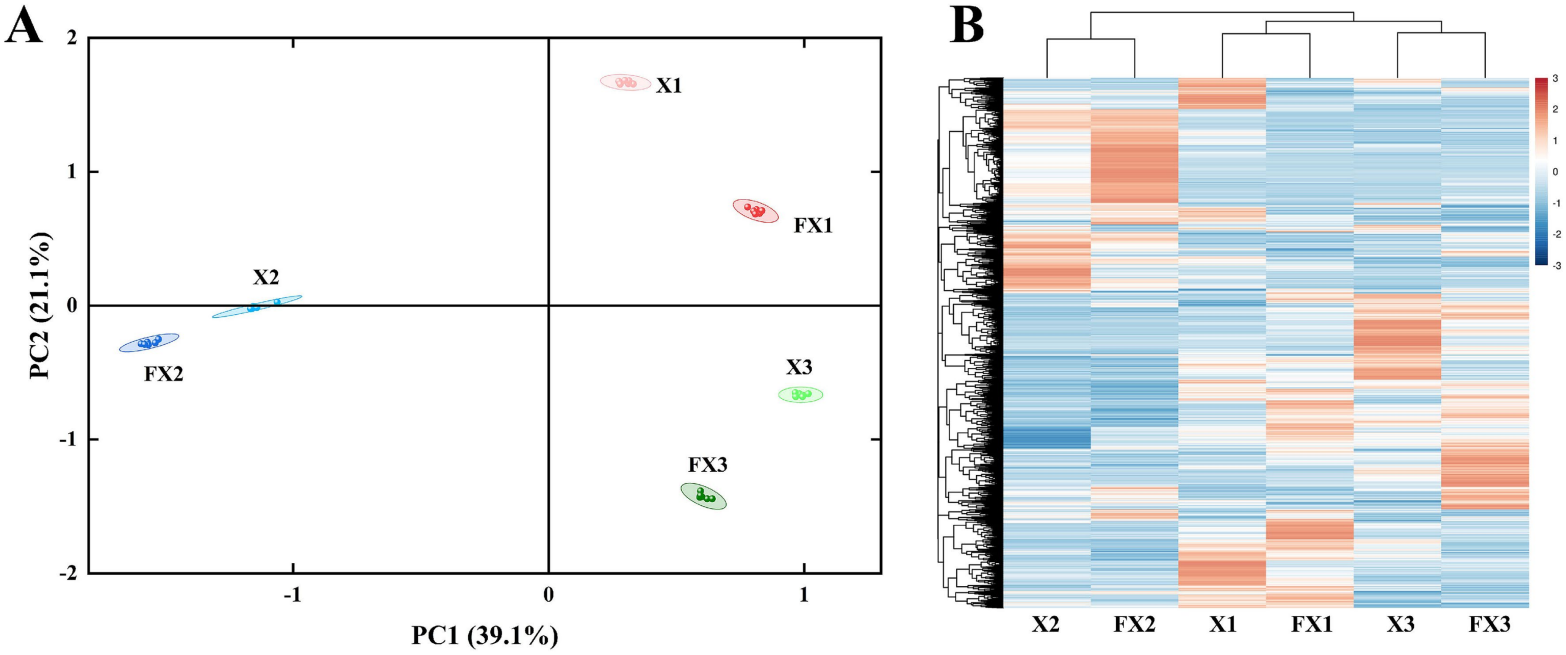
Multivariate analysis of metabolite profiles in FTLs before and after fermentation. **(A)** Principal component analysis (PCA) illustrating the metabolic distribution of different-grade FTL samples before and after fermentation. **(B)** Hierarchical clustering heatmap of metabolite expression, highlighting similarity patterns among samples based on their metabolic profiles.

To delineate compositional shifts in metabolites during fermentation, we performed PCA and hierarchical clustering of metabolite intensities across all samples (Figure 5). As shown in Figure 5A, PCA clearly separated samples by both grade and treatment state. PC1 and PC2 explained 39.1 and 21.1% of the total variance, respectively, with a cumulative contribution of 60.2%. In PC space, the pre-fermentation groups (X1, X2, X3) were already well differentiated, indicating that the intrinsic grade exerts a dominant influence on the baseline metabolic background. After fermentation, the corresponding groups (FX1, FX2, FX3) shifted markedly along both PC1 and PC2, demonstrating substantial fermentation-induced remodeling of the metabolic landscape; however, each FX group remained closest to its cognate X group, implying that fermentation reshapes but does not override the grade-defined architecture.

The heat-map–based hierarchical clustering of metabolite expression (Figure 5B) corroborated this pattern. Samples exhibited highly consistent grade-wise aggregation: X1 clustered with FX1, X2 with FX2, and X3 with FX3. Thus, although fermentation introduced fluctuations in the abundance of specific metabolites, the overall metabolic profile remained stable within the grade framework. In other words, the initial grade exerts a determinant effect on metabolic composition, whereas fermentation acts as a functional fine-tuning superimposed on that baseline. From the perspective of global metabolome structure, fermentation behaves more as a layered modulation than a replacement of the primary determinant.

This dual control—grade determining, fermentation modulating—provides a metabolic rationale for the preservation of flavor characteristics in fermented FTLs and reveals the continuity of high-grade profiles during processing, which is beneficial for maintaining product flavor consistency. More broadly, these findings illuminate the balance between stability and plasticity in metabolic networks during fermentation and point to promising directions for subsequent identification of key differential metabolites and pathways.

### 3.6 Identification of differential metabolites and enrichment analysis of key metabolic pathways

**Figure 6:**
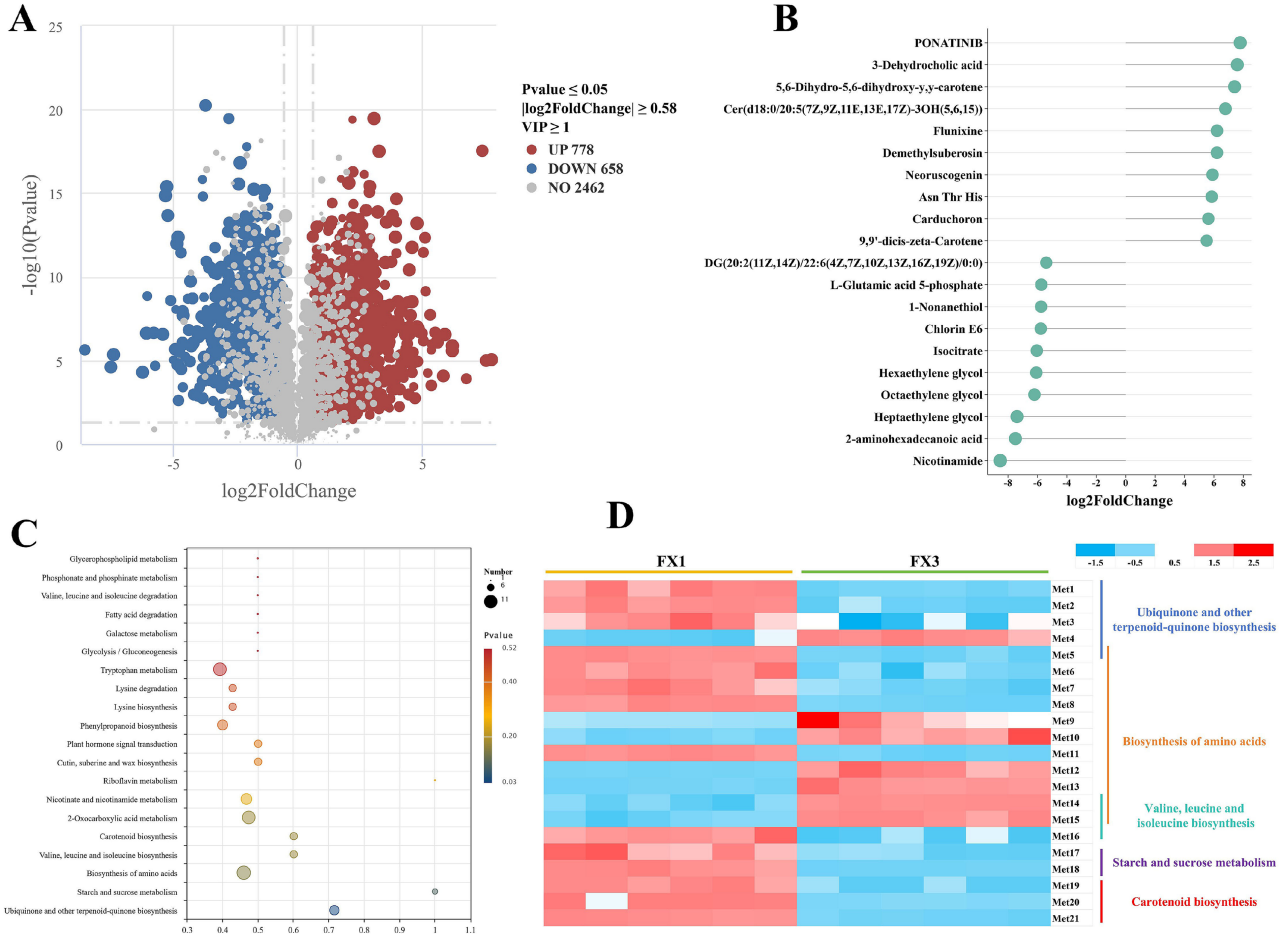
Identification of differential metabolites and enrichment of functional pathways between FX1 and FX3 groups. **(A)** Volcano plot of differential metabolites. **(B)** Bar chart of the top 10 upregulated and top 10 downregulated metabolites ranked by absolute log₂FC. **(C)** KEGG pathway enrichment bubble plot. **(D)** Heatmap of 21 representative differential metabolites involved in the top five significantly enriched KEGG pathways. Met1-Met21 represent the following compounds: alpha-tocotrienol, 2-polyprenylphenol, (1R,6R)-6-hydroxy-2-succinylcyclohexa-2,4-diene-1-carboxylate, hydroxyphenyllactic acid, chorismate, oxoadipic acid, L-proline, N-acetyl-L-glutamate 5-semialdehyde, 3-dehydroquinic acid, isocitrate, L-glutamic acid 5-phosphate, 2,6-diaminoheptanedioic acid, L-saccharopine, L-leucine, alpha-isopropylmalate, 2-isopropylmaleate, trehalose, D-fructose, 9,9′-dicis-zeta-Carotene, 15-cis-phytoene, and (+/−)-abscisic acid.

To dissect post-fermentation metabolic differences between grades, we conducted a multi-level differential analysis comparing FX1 and FX3 (Figure 6). The volcano plot (Figure 6A) identified 1,436 differential metabolites under the criteria VIP ≥ 1, *p* < 0.05, and |log₂FC| ≥ 0.58, of which 778 were significantly higher in FX3 and 658 were higher in FX1, indicating pronounced grade-dependent divergence in metabolic composition after fermentation.

The top 20 metabolites ranked by absolute log₂FC are shown in Figure 6B. Features enriched in FX1 included ponatinib, 3-dehydrocholic acid, 5,6-dihydro-5,6-dihydroxy-y,y-carotene, and demethylsuberosin. By contrast, FX3 was enriched in nicotinamide, 2-aminohexadecanoic acid, L-glutamic acid 5-phosphate, and isocitrate.

KEGG pathway enrichment (Figure 6C) revealed that differential metabolites were mainly associated with ubiquinone and other terpenoid-quinone biosynthesis, starch and sucrose metabolism, valine, leucine and isoleucine biosynthesis/degradation, amino acid biosynthesis, and carotenoid biosynthesis. Based on the five most significant pathways, 21 key differential metabolites were selected for heat-map visualization (Figure 6D). Most of these—D-fructose, trehalose, (+/−)-abscisic acid, chorismate, L-proline, and *α*-tocotrienol, among others—showed higher levels in FX1, indicating that high-grade FTLs develop a richer profile of secondary and signaling metabolites during fermentation. In comparison, only seven metabolites were higher in FX3—hydroxyphenyllactic acid, 3-dehydroquinic acid, isocitrate, 2,6-diaminoheptanedioic acid, L-saccharopine, L-leucine, and α-isopropylmalate—reflecting a distinct metabolic emphasis in the low-grade group.

### 3.7 Correlation analysis between microbial genera and metabolites

**Figure 7:**
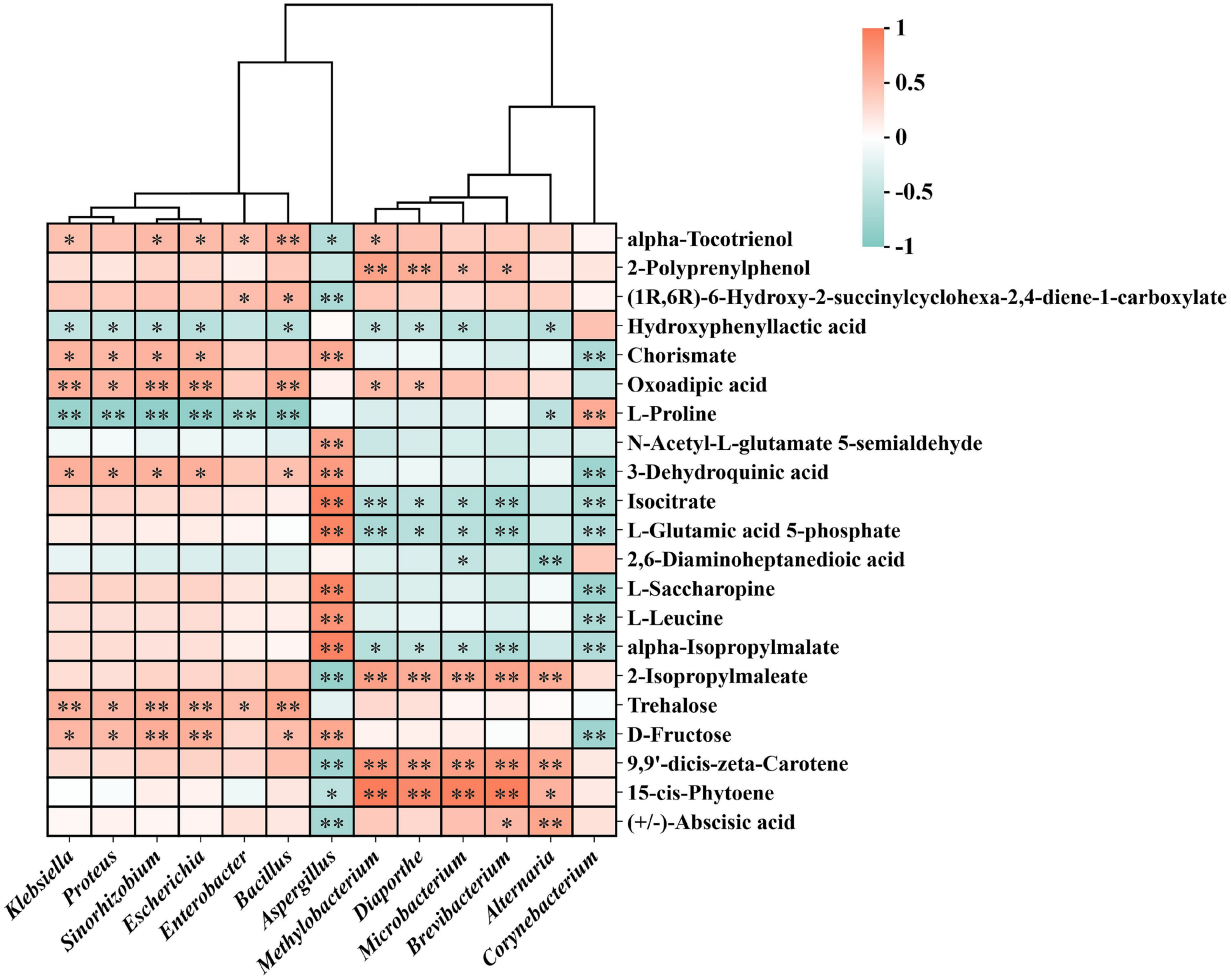
Pearson correlation heatmap between dominant microbial genera and key metabolites during FTLs fermentation. The heatmap presents Pearson correlations between dominant microbial genera identified through LEfSe analysis and 21 differential metabolites from five significantly enriched KEGG pathways. Darker colors indicate stronger correlations. The hierarchical clustering highlights potential microbe–metabolite interaction networks underlying the fermentation process of FTLs.

To explore potential interactions between dominant microbial genera and key metabolites, we constructed a hierarchically clustered Pearson correlation heat map (Figure 7) using the 13 differential genera identified by LEfSe (Figure 3A) and 21 representative metabolites drawn from the five KEGG-enriched pathways (Figure 6D). Several genera—*Bacillus*, *Enterobacter*, and *Klebsiella*—showed significant positive correlations with carotenoids, sugars, and lipophilic metabolites, exemplified by trehalose, 9,9′-dicis-zeta-carotene, and (+/−)-abscisic acid. In contrast, *Corynebacterium* (enriched in FX3), as well as *Brevibacterium* and *Alternaria* (enriched in FX1), exhibited negative correlations with multiple amino acid, aromatic, and sugar-related metabolites.

## 4 Discussion

Fermentation is a traditional yet dynamic biotransformation process that plays a vital role in enhancing the flavor, aroma, and chemical complexity of FTLs (Qiao et al., 2024; Zhang et al., 2025a). Microorganisms, as the core biocatalysts, can mediate hydrolysis, oxidation, glycosylation, deglycosylation, and other reactions to drive the remodeling of both primary and secondary metabolites (Zeng et al., 2023; Zhang L. et al., 2025). However, the interplay between raw material grade and microbial metabolic performance remains insufficiently understood. By integrating metagenomic and untargeted metabolomic analyses, the present study elucidated the structural and functional shifts of microbial communities before and after fermentation across FTLs of different grades, as well as their coupling with metabolic remodeling. This revealed both general grade-associated patterns and deviations from expected grade trends.

In terms of alpha diversity, the changes in the medium-grade group (FX2 vs. X2) after fermentation were less pronounced compared to the high-grade (FX1) and low-grade (FX3) groups, suggesting possible incomplete fermentation or suboptimal activation of functional microbial taxa in FX2. Previous studies have indicated no significant overall differences in alpha diversity among grades under standard fermentation conditions (Zhang et al., 2024a,b), while others observed higher Shannon and Simpson indices in high-grade samples (Xing et al., 2024). The limited changes in FX2 may stem from weak microbial colonization capacity, low activity of key enzymatic systems, or unfavorable microenvironmental conditions such as moisture and pH. These findings suggest that for medium-grade materials, functional microbial inoculation or parameter optimization may be necessary to unlock fermentation potential and achieve comparable outcomes to high- and low-grade FTLs.

Regarding community composition (Figure 1), *Proteobacteria*, *Firmicutes*, and *Ascomycota* were dominant phyla, although previous studies have reported different dominant taxa depending on origin, variety, and fermentation stage (Zhang et al., 2024a; Liu et al., 2022; Si et al., 2023). At the species level (Figure 1D), *Corynebacterium ammoniagenes* reached approximately 50% abundance in FX3, forming a highly dominant structure. This taxon was already present at the X3 stage and may have expanded rapidly during fermentation due to its strong environmental adaptability. Although it can produce umami-related nucleotides via purine/pyrimidine metabolism (Kamada et al., 2003), it also generates substantial ammonia, which may contribute to undesirable odors and compromise sensory quality (León-Vallejo et al., 2019). In contrast, FX1 maintained a more balanced and diverse microbial network post-fermentation, conducive to aroma synthesis and quality improvement (Xing et al., 2023). It is important to note that a higher number of functional pathways does not necessarily equate to superior sensory quality. Although FX3 showed enriched potential in pathways such as carbon and nitrogen metabolism and environmental adaptation (Zhang et al., 2024b), their positive impact on flavor is uncertain if skewed toward stress maintenance rather than aroma generation. This is consistent with the findings of Hu et al. (2023), who noted that moderate levels (<10%) of *Corynebacterium* may benefit quality, whereas excessive levels can be detrimental.

Metabolomic analysis revealed distinct post-fermentation metabolic profiles across grades (Figures 5, 6). FX1 was enriched with metabolites bearing aromatic rings or carotenoid skeletons, associated with sugar metabolism, carotenoid biosynthesis, and signaling regulation (Ren et al., 2024; Jiang et al., 2024a). These findings, along with Geng et al. (2023), suggest that high-grade FTLs tend to accumulate flavor precursors, antioxidants, and complex secondary metabolites. Conversely, FX3 featured metabolites related to energy metabolism, amino acid metabolism, and stress responses (Ren et al., 2023; Song et al., 2024). The pronounced metabolic divergence highlights the stronger aroma potential and quality attributes of high-grade FTLs and provides a metabolomic basis for grade discrimination and biomarker discovery (see Figures 6B,D).

Furthermore, microbial-metabolite correlation analysis (Figure 7) revealed potential causal links and regulatory nodes. *Bacillus*, *Enterobacter*, and *Klebsiella*, enriched in FX1, were positively correlated with carotenoids, sugars, and lipophilic metabolites such as trehalose, 9,9′-dicis-zeta-carotene, and (+/−)-abscisic acid. These compounds are closely associated with color development, flavor precursor accumulation, and signal transduction. This suggests that these genera may facilitate the construction of flavor-related networks through glycosidic hydrolysis, substrate transport, and secondary metabolic coordination (Wei et al., 2024). Previous reports by Gong et al. (2023) and Jiang et al. (2024b) showed that *Bacillus* strains isolated from tobacco leaves can activate endogenous enzymes, promoting the degradation of macromolecules like starch and proteins, thereby enhancing aroma release. This aligns with the idea that microbially mediated transformations such as glycosylation/deglycosylation and esterification/de-esterification can drive the development of flavor characteristics, thus supporting the superior sensory performance of FX1.

Conversely, *Corynebacterium* in FX3, along with *Brevibacterium* and *Alternaria* in FX1, were negatively correlated with multiple amino acid, aromatic, and sugar metabolites, suggesting that under certain fermentation conditions, these taxa may suppress the synthesis of key flavor compounds via metabolic competition, resource consumption, or signaling interference (Wu et al., 2024a,b). Notably, although *Aspergillus* was the dominant fungal genus in FX3 and positively correlated with many metabolites, it may play a dual role: while it is a known cause of spoilage during air curing and fermentation (Wu et al., 2024a; Fu et al., 2024), it can also degrade macromolecules like proteins and starch, reducing off-flavors and bitterness and promoting flavor precursor formation under controlled conditions (Fang et al., 2024; Jia et al., 2025). Therefore, its application is context-dependent: under regulated environments, *Aspergillus* may serve as a functional candidate for directional fermentation, whereas in uncontrolled settings, it poses a spoilage risk. The standardized use of this “double-edged sword” genus may offer a viable strategy to enhance the flavor quality of low-grade FTLs.

At the pathway level, FX3 was more oriented toward basic metabolic maintenance and environmental response (Liu et al., 2021), whereas FX1 was enriched in pathways related to signal transduction, ABC transporters, material transport and catabolism, and lipid metabolism, which are more closely associated with quality (Wu et al., 2023). This indicates that high-grade FTLs are more likely to establish a functionally integrated network involving multidimensional regulation, high-throughput transport, and complex synthesis, facilitating the coordinated accumulation of pigments, sugars, and aroma-related metabolites. In contrast, although low-grade FTLs exhibit adequate basic metabolism and stress-response functions, their contribution to flavor complexity and stability may be limited by the ecological imbalance and functional bias of their microbial communities.

The grade-specific patterns of microbial-metabolite interactions during fermentation suggest that the relationship between grade and fermentation performance is not strictly linear. High-grade samples, with greater community diversity and enrichment in signal transduction, transport, and secondary metabolism pathways, may foster a microecological environment conducive to aroma development, enhancing sensory attributes such as color, aroma, and smoothness. Conversely, low-grade samples may display metabolic activity in basic pathways but are constrained by single-dominant community structures and stress-oriented functions, potentially limiting flavor richness and stability. Notably, the limited change observed in FX2 suggests that raw material grade alone is not the sole determinant of fermentation performance. Factors such as microbial colonization capacity, enzymatic expression, and environmental parameters may play more decisive roles. Therefore, activating and maintaining a functionally complementary and ecologically stable microbe-metabolite network may be more critical to product quality than grade alone.

Nevertheless, this study acknowledges several limitations. Firstly, although tobacco leaf grading was based on the YC/T 588—2021 standard, previous studies (Zhang et al., 2024a) have noted that current grading practices rely heavily on sensory evaluation, which can be subjective and prone to evaluator variability and workload limitations, possibly leading to inconsistent quality due to grade mixing. Secondly, the fermentation process is considered proprietary to each enterprise and varies in duration from several days to weeks. In this study, industrial fermentation lasted 1 week, with relatively low initial moisture content, which may have limited microbial colonization and activity, particularly in the FX2 group. Thirdly, although GC–MS-based detection excels in characterizing volatile flavor compounds, many tobacco-related aromas arise from the conversion of non-volatile precursors. Thus, untargeted UHPLC–MS/MS profiling of non-volatile metabolites remains essential for a comprehensive understanding of flavor-relevant metabolic shifts during fermentation.

Future efforts could focus on: (i) targeted domestication and inoculation of functional microbes for medium/low-grade samples; (ii) fine-tuned control of key fermentation parameters such as moisture, temperature-humidity fluctuation, and pH; and (iii) regulation of core enzymatic systems (e.g., glycosidases, transporters, lipid synthases and lyases) to precisely guide fermentation pathways and achieve consistent quality enhancement across different FTL grades.

## 5 Conclusion

This study systematically characterized the dynamic changes in microbial communities and metabolomic profiles of FTLs of different grades before and after fermentation. Metagenomic analysis revealed that distinct microbial structures were already established at the onset of fermentation and were further modulated by the process, with high-grade FTLs enriched in genera such as *Bacillus*, *Escherichia*, and *Alternaria*, which are associated with macromolecule degradation, aroma precursor biosynthesis, and signal transduction. In contrast, excessive *Corynebacterium* in low-grade samples may produce off-flavors such as ammonia, adversely affecting product quality. Untargeted metabolomics indicated significant fermentation-induced accumulation of metabolites, particularly those involved in “amino acid biosynthesis,” “starch and sucrose metabolism,” and “carotenoid biosynthesis.” PCA and hierarchical clustering demonstrated grade-dependent continuity in metabolic profiles, while correlation analysis revealed strong associations between key metabolites and dominant microbial genera. Overall, the initial grade of FTLs plays a decisive role in directing the co-evolution of microbial and metabolic networks during fermentation, providing theoretical insight into flavor formation mechanisms and practical guidance for optimizing leaf grading and improving cigar product consistency.

## Data Availability

The data presented in the study are deposited in the NCBI Sequence Read Archive (https://www.ncbi.nlm.nih.gov/sra), accession number PRJNA1279796.

## Footnotes

^1^ https://www.chiplot.online/
